# BMC Family Practice reviewer acknowledgement 2014

**DOI:** 10.1186/s12875-015-0229-6

**Published:** 2015-02-22

**Authors:** Magdalena Morawska

**Affiliations:** BioMed Central, Floor 6, 236 Gray’s Inn Road, London, WC1X 8HB United Kingdom

## Abstract

The editors of BMC Family Practice would like to thank all of our reviewers who have contributed to the journal in Volume 15 (2014).

**Aase Aamland**

Norway

**Marcel Adriaanse**

Netherlands

**Girdhar Agarwal**

India

**Afaf Al-Adsani**

Kuwait

**Sarah Alderson**

UK

**Jill Allison**

Canada

**Foteini Anastasiou**

Greece

**Annika Anden**

Sweden

**Robyn Andersen**

USA

**Natalie Armstrong**

UK

**David Aron**

USA

**Britt Arrelov**

Sweden

**Megan Arroll**

UK

**Mark Ashworth**

UK

**Marzia Baldereschi**

Italy

**Claire Balmer**

UK

**Michelle Banfield**

Australia

**M John G Bankart**

UK

**Martin Bardsley**

UK

**Elizabeth Barley**

UK

**Alan Barnard South**

Africa

**Christopher Barton**

Australia

**Malcolm Battersby**

Australia

**Miguel Bedolla**

USA

**Abdulbari Bener**

Qatar

**Antje Bergmann**

Germany

**Kurt Bestehorn**

Germany

**Elizabeth Beverly**

USA

**Martin Beyer**

Germany

**Stella Bialous**

USA

**Jade Bilardi**

Australia

**Ugur Bilge**

Turkey

**Markus Bleckwenn**

Germany

**Christian Blickem**

UK

**Karin Blomberg**

Sweden

**Richard Body**

UK

**Fiona Boland**

Ireland

**Stefan Bösner**

Germany

**Sinthia Bosnic-Anticevich**

Australia

**Georg Bosshard**

Switzerland

**Samantha Bracebridge**

UK

**Matthias Briel**

Switzerland

**B.D. Lidewij Broekhuizen**

Netherlands

**Joanna Brooks**

UK

**Carlos Brotons**

Spain

**Scott Brown**

UK

**Lauro Bucchi**

Italy

**Bettina Buecker**

Germany

**Mateja Bulc**

Slovenia

**Heather Burroughs**

UK

**Christopher Burton**

UK

**Amaia Calderón-Larrañaga**

Spain

**John Cape**

UK

**Sandra Capra**

Australia

**Siw Carlfjord**

Sweden

**Concepcion Carratala-Munuera**

Spain

**Melinda Carrington**

Australia

**Jenny Carryer**

New Zealand

**Andrew Carson-Stevens**

UK

**Stephen Carter**

Australia

**Rufus Cartwright**

UK

**Yee-Ling Chang**

Canada

**Victor Chel**

Netherlands

**Liying Chen**

China

**Xiangmei Chen**

China

**Sudeh Cheraghi-Sohi**

UK

**Boon How Chew**

Malaysia

**Jennifer Cleland**

UK

**Deanne Lynn Clouder**

UK

**Alan Clough**

Australia

**Barbara Clyne**

Ireland

**Simon Cocksedge**

UK

**Rubin Cohen**

USA

**Anna Coleman**

UK

**Claire Collins**

Ireland

**Elizabeth Cottrell**

UK

**Peter Alan Coventry**

UK

**Luke Cowie**

UK

**Jesse Crosson**

USA

**Walter Cullen**

Ireland

**Jacqueline Cumming**

New Zealand

**Sara Czaja**

USA

**Olivia Dalleur**

Belgium

**Gianfranco Damiani**

Italy

**Olga Catherina Damman**

Netherlands

**Avril Danczak**

UK

**David Darmon**

France

**Dinny De Bakker**

Netherlands

**Derek De Beurs**

Netherlands

**Anna De Simoni**

UK

**Lore Decoster**

Belgium

**Jean-Marie Degryse**

Belgium

**Richard Dekhuijzen**

Netherlands

**Borislav Dimitrov**

UK

**Geert-Jan Dinant**

Netherlands

**Christa Doerr**

Germany

**Stephan Dombrowski**

UK

**Michael Drazer**

USA

**Jessica Drinkwater**

UK

**James Dunbar**

Australia

**Mark Ebell**

USA

**Marina Economou**

Greece

**R. Erandie Ediriweera De Silva**

Sri Lanka

**Samuel Edwards**

USA

**Petra Elders**

Netherlands

**Yvonne Engels**

Netherlands

**Meirion Evans**

UK

**Hazel Everitt**

UK

**Isabelle Fabbricotti**

Netherlands

**Tom Fahey**

Ireland

**Karen Fairhurst**

UK

**Tomas Faresjo**

Sweden

**Annunziata Faustini**

Italy

**Susan Fisher-Hoch**

USA

**Gerry Fitzgerald**

Australia

**Daniela Fodor**

Romania

**Marjolein Fokkema**

Netherlands

**Nadine Foster**

UK

**Robbie Foy**

UK

**Jessica Fraeyman**

Belgium

**Nick Francis**

UK

**Michael Freitag**

Germany

**Thomas Frese**

Germany

**Tobias Freund**

Germany

**Brona Fullen**

Ireland

**Jeffrey Fuller**

Australia

**Sander Gaal**

Netherlands

**Mark Gabbay**

UK

**Rose Galvin**

Ireland

**Idoia Gaminde**

Spain

**Karen Gardner**

Australia

**Natalie Gauld**

New Zealand

**Marianne Gee**

Canada

**Marloes Gerrits**

Netherlands

**Stephen Gillam**

UK

**Svein Gjelstad**

Norway

**Gerd M. Glaeske**

Germany

**Margaret Glogowska**

UK

**Maciek Godycki-Cwirko**

Poland

**Claire Goodman**

UK

**Aileen Grant**

UK

**Liz Grant**

UK

**Monica Greco**

UK

**David Greenfield**

Australia

**Sheila Greenfield**

UK

**Carlos Grijalva-Eternod**

UK

**Sietske Grol**

Netherlands

**Kevin Grumbach**

USA

**Pilar Guallar-Castillón**

Spain

**Ann_Dorrit Guassora**

Denmark

**Julian Guest**

UK

**Martin Gulliford**

UK

**Michael Gusmano**

USA

**David Hanley**

UK

**Janet Hanley**

UK

**Reina Haque**

USA

**Shamil Haroon**

UK

**Mark Harris**

Australia

**Jo Hart**

UK

**Gill Harvey**

UK

**Andrew Hassell**

UK

**Emma Healey**

UK

**Maggie Hendry**

UK

**Luis Hernandez-Gomez**

Spain

**Cees M.P.M. Hertogh**

Netherlands

**Wolfgang Himmel**

Germany

**Nobutaka Hirooka**

Japan

**Ingibjörg Hjaltadóttir**

Iceland

**Sjoerd Obe Hobma**

Netherlands

**Arno Hoes**

Netherlands

**William Hogg**

Canada

**Monika Hollander**

Netherlands

**Sandra Hollinghurst**

UK

**Tim Holt**

UK

**Emiel Hoogendijk**

Netherlands

**Patricia Hudelson**

Switzerland

**Ben Hudson**

New Zealand

**John Hughes**

UK

**Josabeth Hultberg**

Sweden

**Cheryl Hunter**

UK

**Catherine Hyde**

UK

**David Ikkersheim**

Netherlands

**Steve Iliffe**

UK

**Francesca Innocenti**

Italy

**Liisa Jaakkimainen**

Canada

**Wasim Jafri**

Pakistan

**Justin Jagosh**

Canada

**Nitin Jain**

USA

**Poorva Jain**

UK

**Bhautesh Dinesh Jani**

UK

**Ralf Jendyk**

Germany

**Unn-Britt Johansson**

Sweden

**Sharon Johnston**

Canada

**Ulrike Junius-Walker**

Germany

**Lise Juul**

Denmark

**Umesh Kadam**

UK

**Hanna Kaduszkiewicz**

Germany

**László Kalabay**

Hungary

**Ali Yavuz Karahan**

Turkey

**Konstantinos Karatolios**

Germany

**Margaret Kay**

Australia

**Kathleen Kendall**

UK

**Anthony Kendrick**

UK

**Anne Kennedy**

UK

**Lindsey Kettinger**

USA

**Andreas Klement**

Germany

**C M Frank Kneepkens**

Netherlands

**Sarah Knowles**

UK

**Elias Kondilis**

Greece

**Joke Korevaar**

Netherlands

**Thomas Kötter**

Germany

**Antonis Kousoulis**

UK

**Eszter Kovacs**

Hungary

**Karin Kraft**

Germany

**Lawrence Krakoff**

USA

**Vishwajeet Kumar**

India

**Cindy Lam**

Hong Kong

**Jonathan Lamb**

UK

**Barry Lambe**

Ireland

**Susanne Langer**

UK

**Sarah Larkins**

Australia

**Sarah Larney**

Australia

**Laura Laslett**

Australia

**John Launer**

UK

**Caroline Laurence**

Australia

**Ross Lawrenson**

New Zealand

**M. Barton Laws**

USA

**Thomas Laws**

Australia

**Sally Lawton**

UK

**Natalie Leon**

South Africa

**Jean-Frederic Levesque**

Australia

**Martyn Lewis**

UK

**George Lewith**

UK

**Jennifer Liddle**

UK

**Su May Liew**

Malaysia

**Klaus Linde**

Germany

**Jeffrey Linder**

USA

**Keng-Yin Loh**

Malaysia

**Carl Lombard**

South Africa

**Maja Lopez Hartmann**

Belgium

**Cristina Lopez-Del Burgo**

Spain

**Ramon Luengo-Fernandez**

UK

**Una Macleod**

UK

**Parker Magin**

Australia

**Megan Mahoney**

USA

**Chris Main**

UK

**Lilach Malatskey**

Israel

**Andrew Mallett**

Australia

**Rebecca Malouin**

USA

**Kirsti Malterud**

Norway

**David Margolius**

USA

**Laura Marlow**

UK

**Yolanda V Martinez**

UK

**Rohini Mathur**

UK

**Margaret Maxwell**

UK

**Lindsay Mayberry**

USA

**Kerry Mcbrien**

Canada

**Julie Mcdonald**

Australia

**Ruth Mcdonald**

UK

**Darryl Mcgill**

Australia

**Eileen Mckinlay**

New Zealand

**Brian Mckinstry**

UK

**Gary Mclean**

UK

**Kate Mclintock**

UK

**Hasse Melbye**

Norway

**René JF Melis**

Netherlands

**Eero Merilind**

Estonia

**Anastasia Mihailidou**

Australia

**Antje Miksch**

Germany

**Ingrid Miljeteig**

Norway

**Linda Milnes**

UK

**Sherina Mohd Sidik**

Malaysia

**Michael Moore**

UK

**Patrick Moran**

Ireland

**Leila B Moreira**

Brazil

**John Morris**

Ireland

**Val Morrison**

UK

**Richard Morriss**

UK

**Anne Mullin**

UK

**Maaike Muntinga**

Netherlands

**Mark Murphy**

Ireland

**Lucio Naccarella**

Australia

**Yusuf Nagree**

Australia

**Susan Nancarrow**

Australia

**Stefan Neuner-Jehle**

Switzerland

**Wendy Nicklin**

Canada

**Staffan Nilsson**

Sweden

**Janneke Noordman**

Netherlands

**Elaine Oconnell Francischetto**

UK

**Amy O’Donnell**

UK

**Ellis Owusu-Dabo**

Ghana

**Kayihan Pala**

Turkey

**Mary Palmer**

USA

**Sukhmeet Panesar**

UK

**Maria Grazia Pascucci**

Italy

**Mahendra Patel**

UK

**Neesha Patel**

UK

**Avinash Patwardhan**

USA

**Winifred Paulis**

Netherlands

**Rupert Payne**

UK

**David Pearson**

USA

**Mark Pearson**

UK

**Marija Petek-Ster**

Slovenia

**Sarah Peters**

UK

**Frank Peters-Klimm**

Germany

**Lars Peterson**

USA

**Patrizio Pezzotti**

Italy

**Tamar Pincus**

UK

**Luca Pingani**

Italy

**Edith Poku**

UK

**Rani Polak**

USA

**Alison Pooler**

UK

**Kevin Pottie**

Canada

**Egle Price**

UK

**Marijn Alice Prins**

Netherlands

**Maria Pufulete**

UK

**Waris Qidwai**

Pakistan

**Virginia Quinn**

USA

**Jennifer Quint**

UK

**Imran Rafi**

UK

**Vivian R Ramsden**

Canada

**Sudhaker D Rao**

USA

**Freya Rasschaert**

Belgium

**Paivi Rautava**

Finland

**Srinevas Reddy**

USA

**Ruth Reed**

UK

**Anthony Reeder**

New Zealand

**Carole Reeve**

Australia

**Joanne Reeve**

UK

**David Reeves**

UK

**Jim Reid**

New Zealand

**Suzanne Richards**

UK

**John Richmond**

UK

**Janez Rifel**

Slovenia

**Pasquale Roberge**

Canada

**Helen Rodgers**

UK

**Anne Elizabeth Rogers**

UK

**Stephen Rogers**

UK

**Martin Roland**

UK

**Irene Romera**

Spain

**Marianne Rosendal**

Denmark

**Danica Rotar-Pavlic**

Slovenia

**Bruno Rushforth**

UK

**Guy Rutten**

Netherlands

**Dirk Ruwaard**

Netherlands

**Christina Ryborg**

Denmark

**Maria Salinas**

Spain

**Chris Salisbury**

UK

**Gil Salles**

Brazil

**Lipika Samal**

USA

**Guillermo Sanchez**

USA

**Stefan Sauerland**

Germany

**Ingmar Schaefer**

Germany

**Peter Schattner**

Australia

**Guido Schmiemann**

Germany

**Nils Schneider**

Germany

**Birgitte Schoenmakers**

Belgium

**Siebrig Schokker**

Netherlands

**Sarah Hudson Scholle**

USA

**Neha Sekhri**

UK

**Miho Sekimoto**

Japan

**Vanessa Selak**

New Zealand

**Frederic Selck**

USA

**Oliver Senn**

Switzerland

**Vincent Setlhare**

Botswana

**Laura Shallcross**

UK

**Donna Shelley**

USA

**Dong-Soo Shin**

South Korea

**Judith Sim**

UK

**Rosemary Simmonds**

UK

**Surinder Singh**

UK

**Aloysius Niroshan Siriwardena**

UK

**Jeffrey Sisler**

Canada

**Iain Small**

UK

**Nicola Small**

UK

**Frank Smeenk**

Netherlands

**Daniel Smith**

UK

**Michael Smith**

UK

**Susan Smith**

Ireland

**Sarah Smithson**

UK

**Andreas Sonnichsen**

Germany

**Lena Spangenberg**

Germany

**Sharon Spooner**

UK

**Anthony Staines**

Ireland

**Renee Stalmeijer**

Netherlands

**Lindsay Stead**

UK

**Katharina Viktoria Stein**

Austria

**Gregory Stevens**

USA

**Fiona Stevenson**

UK

**Joanna Stewart**

New Zealand

**Erin Strumpf**

Canada

**Naeti Suksomboon**

Thailand

**Frank Sullivan**

UK

**Frank Sullivan**

Canada

**Patricia Sunaert**

Belgium

**Elisabeth Svensson**

Sweden

**Geeta Swamy**

USA

**Kevin Sweet**

USA

**Jane Taggart**

Australia

**Rosemary Tate**

UK

**Athina Tatsioni**

Greece

**Anna Taylor**

UK

**Clare Taylor**

UK

**Berend Terluin**

Netherlands

**Erik Teunissen**

Netherlands

**Gudrun Theile**

Switzerland

**Markus Themessl-Huber**

UK

**Trevor Thompson**

UK

**Maria Thomson**

USA

**Philip Tideman**

Australia

**Petter Tinghog**

Sweden

**Christiana Titaley**

Indonesia

**Gail Todd**

South Africa

**Barbara Tomenson**

UK

**Sarah Tonkin-Crine**

UK

**Erik Trell**

Sweden

**Ioanna Tsiligianni**

Greece

**Katrina Turner**

UK

**Nikolaos Tzanakis**

Greece

**Jenny Ure**

UK

**Rodolfo Valdez**

USA

**Kees Van Boven**

Netherlands

**Alike Van Der Velden**

Netherlands

**Jouke Van Der Zee**

Netherlands

**Liset Van Dijk**

Netherlands

**Susan Van Dijk**

Netherlands

**Sonja Van Dillen**

Netherlands

**Harm Van Marwijk**

Netherlands

**Paul Van Royen**

Belgium

**Jessie Vanswearingen**

USA

**Peter Vedsted**

Denmark

**Theo Verheij**

Netherlands

**Wim V Verstappen**

Netherlands

**Shlomo Vinker**

Israel

**Olaf Von Dem Knesebeck**

Germany

**Jeremy Walker**

UK

**Qing Wan**

Australia

**Robert Ware**

Australia

**Alex Warner**

UK

**Patrick Waterson**

UK

**Li Wei**

UK

**Sara Willems**

Belgium

**Jack Williams**

Canada

**Kate Williams**

UK

**Andrew Wilson**

UK

**Adam Windak**

Poland

**Jennifer Wingham**

UK

**Ron Winkens**

Netherlands

**Nahathai Wongpakaran**

Thailand

**Jenny Woodman**

UK

**Edwin Wouters**

Belgium

**John Yaphe**

Portugal

**Sarah Yardley**

UK

**Kathleen Yost**

USA

**Zhaokang Yuan**

China

**M Osman Yusuf**

Pakistan

**Chengchao Zhou**

China

**Manuel Zorzi**

Italy

**Nicholas Zwar**

Australia

**Sa’Ed Zyoud**

Palestinian Territory

